# Green surgery: a systematic review of the environmental impact of laparotomy, laparoscopy, and robotics

**DOI:** 10.1007/s13304-025-02221-1

**Published:** 2025-05-21

**Authors:** Miguel F. Cunha, João Cunha Neves, Joana Roseira, Gianluca Pellino, Pedro Castelo-branco

**Affiliations:** 1 Surgery Department, Colorectal Surgery, Algarve Local Health Unit, Portimão, Portugal; 2 Faculty of Medicine and Biomedical Sciences (FMCB), University of Algarve, Faro, Portugal; 3 Gastroenterology Department, Algarve Local Health Unit, Portimão, Portugal; 4 Colorectal Surgery, Vall d’Hebron University Hospital, Universitat Autonoma de Barcelona UAB, Barcelona, Spain; 5 Algarve Biomedical Center (ABC), University of Algarve, Faro, Portugal; 6 Algarve Biomedical Center Research Institute (ABC-RI), University of Algarve, Faro, Portugal

**Keywords:** Sustainability, Carbon footprint, Surgery, Open surgery, Minimally invasive surgery, Surgical outcomes

## Abstract

Surgery is the most energy-intensive healthcare sector, but data on the environmental impact of abdominal surgical techniques are limited. This systematic review aims to identify the most sustainable approach among open, laparoscopic, and robotic surgeries. We searched MEDLINE, Cochrane, and Web of Science databases (inception to March 2024) for studies on the carbon footprint of abdominal surgery, focusing on carbon dioxide equivalents (CO_2e_) or CO_2_ emissions. The Joanna Briggs Institute checklist was used to assess bias. (PROSPERO: 298486). Of 2155 records, eight cohort studies were included, showing low to moderate risk of bias but high heterogeneity. Two studies on hysterectomy found robotic surgery had the highest carbon footprint (12.0–40.3 kgCO_2e_) compared to laparoscopic (10.7–29.2 kgCO_2e_) and open surgery (7.1–22.7 kgCO_2e_). Another study found laparoscopic prostatectomy produced more emissions than robotic surgery (59.7 vs. 47.3 kgCO_2e_) due to higher disposable devices, surgery time and length of stay. Single-use devices in laparoscopic cholecystectomy emitted more CO_2e_ than hybrid devices (7.194 vs. 1.756 kgCO_2e_). CO_2_ used in minimally invasive surgery had negligible environmental effects (0.9 kgCO_2e_). Qualitative subgroup analyses revealed significant differences between surgery types and measurement methodologies, contributing to data heterogeneity. Minimally invasive surgeries often have higher carbon footprints due to disposable tools and waste. However, one study showed robotic surgery may reduce the overall environmental impact by shortening hospital stays. Due to methodological heterogeneity across studies, definitive conclusions remain limited. Standardized life-cycle assessment methodologies and inclusion of clinical outcomes in future studies are urgently needed to clarify the environmental sustainability of surgical practices.

**Supplementary Information:**

The online version contains supplementary material available at 10.1007/s13304-025-02221-1.

## Introduction

Climate change represents a current environmental and public health threat [1]. Healthcare systems are responsible for about 10% of all greenhouse gases emissions in the United States of America and are directly responsible for global warming [1–3]. Due to its broad and complex nature, surgery is one of the main contributors to healthcare’s carbon footprint [4]. However, data on the environmental impact of different surgeries, approaches, devices, and materials is still scarce [5]. As a high-throughput speciality, implementing measures towards a more sustainable surgical practice needs further improvement.

Minimally invasive surgery has recently become widespread [6]. Within abdominal surgery, laparoscopy and robotics approaches are considered minimally invasive[7]. Compared with the open approach, minimally invasive techniques have multiple advantages, such as smaller incisions, less intra-operative blood loss, less post-operative pain, earlier oral intake, shorter hospital stays, and faster recovery [8].

We aimed to perform a systematic review to identify the most environmentally sustainable approach in abdominal surgery (open, laparoscopic, or robotics) and its predictors. Additionally, we sought to evaluate whether clinical outcomes were impaired despite the adoption of sustainable measures.

## Methods

A systematic review was conducted following the Preferred Reporting Items for Systematic Reviews and Meta-Analyses (PRISMA) statement guidelines [9].

After ensuring no similar review was registered in The International Prospective Register of Systematic Reviews (PROSPERO), our systematic review protocol details were registered (ID: 298486).

### Search strategy

A systematic search was conducted in MEDLINE/PubMed, CENTRAL/Cochrane, and Web of Science from inception to the 1 st of March 2024. The following keywords or medical subject heading (MeSH) terms, combined with Boolean logical operators, were initially used: “Surgery”, “Laparotomy Surgery”, “Laparoscopy Surgery”, “Robotic Surgery”, “Sustainability”, “Carbon Footprint”, “Environmental Sustainability”, “Outcomes”, “Clinical Outcomes”. This search yielded almost no information concerning clinical outcomes and surgical environmental carbon footprint. As so, a final string deleting these MeSH terms (“Outcomes” and “Clinical Outcomes”) was used (Supplementary 1). No language restrictions were applied.

### Inclusion and exclusion criteria

Studies assessing the environmental impact of abdominal surgery approaches (open, laparoscopic or robotics) were included. All cohort studies enrolling either adult or pediatric patients and studying the environmental impact of abdominal surgery were also eligible. For purposes of metric standardization of the environmental impact, only studies presenting final results of the carbon footprint as carbon dioxide equivalents (CO_2e_) or carbon dioxide (CO_2_) emissions were included. Whenever this data was available, a sub-analysis of the surgical clinical outcomes was performed. Exclusion criteria were as follows: 1) systematic reviews, narrative reviews, animal and in vitro studies, guidelines, editorials, and protocols; 2) studies enrolling patients submitted to non-abdominal surgeries; 3) studies not specifying the surgical approach; 4) studies presenting final carbon footprint results in non-standard measures; 5) studies discussing environmental sustainability concerning sectors other than the healthcare.

### Data evaluation and extraction

Rayyan software for systematic review [10] was used for title and abstract screening after duplicate manual exclusion. Two independent junior reviewers (M.C. and J.C.N.) screened titles and abstracts, and a third senior reviewer (G.P.) solved the conflicts. Afterwards, the same approach was used for full-text manuscripts’ screening and selection (M.C, J.C.N and G.P). In the end, the following information was collected: author, year of publication, methodology, main aim, study duration, number of included patients/surgeries, surgical approach (open and minimally invasive—laparoscopic and robotic), type of surgery/speciality (Ex. hysterectomy), environmental impact metric, outcome, and study conclusion.

### Data quality assessment

The authors (M.C and J.C.N) assessed the studies'methodological quality using the Joanna Briggs Institute (JBI) Critical Appraisal Checklist for cohort studies [11]. The eleven questions that compose the JBI checklist for cohort studies had four possible responses: Yes (the criteria are clearly identifiable through the report description); Unclear (the criteria are not clearly identified in the report); No (the criteria failed to be applied appropriately); N/A (non-applicable) [11]. Two authors (M.C. and J.C.N) independently answered each of the eleven questions and evaluated the quality of each study. In case of disagreement, the study was evaluated by the senior authors (G.P. and P.CB.), and the discrepancy was solved by mutual agreement. Each study was then classified into one of the following categories: low risk of bias (> 60% Yes on JBI); moderate risk of bias (40% < Yes on JBI < 60%); high risk of bias (< 40% of Yes on JBI) [11].

### Statistic considerations

A descriptive analysis was performed. Descriptive data were presented as mean and standard deviation or median and interquartile range. Categorical data were expressed as proportions and percentages.

## Results

**Fig. 1:**
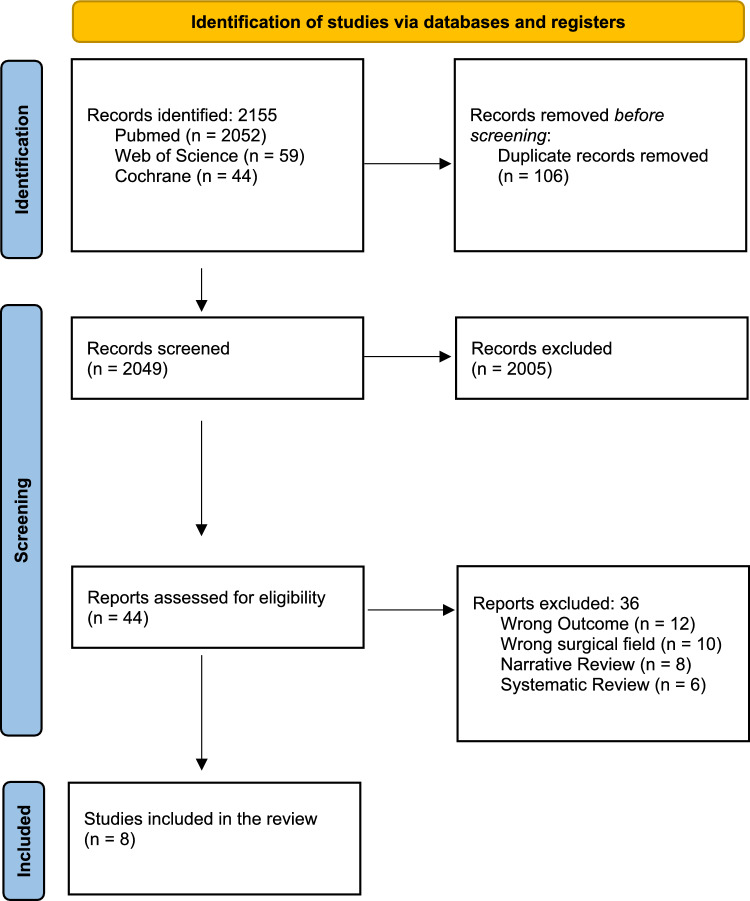
PRISMA diagram

A total of 2155 records were identified, and 106 were manually deleted after a duplicate check. Two thousand forty-nine records were screened for title and abstract, and forty-four were selected for full-text review. In the end, thirty-six papers were excluded (wrong outcome, n = 12; not reporting on abdominal surgery, n = 10; wrong design, n = 14), and eight articles were included in the systematic review (Fig. 1).

### Quality assessment

Six studies were classified as having a low risk of bias, and two as having a moderate risk of bias **(**Table 1**).** The higher risk of bias was mainly driven by the study's confounding factors, as measures to minimize this issue were not disclosed in all but one study [12].

**Table 1:** Risk of bias according to the JBI checklist for cohort studies

Study	Q*1	Q*2	Q*3	Q*4	Q*5	Q*6	Q*7	Q*8	Q*9	Q*10	Q*11	% YES	RISK OF BIAS**
Gilliam et al [16]	X	X	Y	X	X	Y	Y	Y	Y	N/A	Y	55%	Moderate
Power et al [17]	Y	Y	U	X	X	Y	Y	Y	Y	N/A	U	55%	Moderate
Cassandra et al (2014)	Y	Y	Y	Y	X	Y	Y	Y	Y	N/A	X	73%	Low
Woods et al [18]	Y	Y	Y	Y	X	Y	Y	Y	Y	N/A	Y	82%	Low
Cassandra et al [14]	Y	Y	Y	Y	X	Y	Y	Y	Y	N/A	Y	82%	Low
Rizan et al [12]	Y	Y	Y	Y	Y	Y	Y	Y	Y	N/A	Y	91%	Low
Pastorie et al (2022)	Y	Y	Y	Y	X	Y	Y	Y	Y	N/A	Y	82%	Low
Ramani et al (2023)	Y	Y	Y	Y	Y	Y	Y	Y	Y	N/A	Y	91%	Low

^*^Question of the Joanna Briggs Institute (JBI) Critical Appraisal Checklist for cohort study

^**^ High risk of bias was considered if < 40% of Yes on JBI; moderate risk of bias was considered if > 40% and < 60% of Yes on JBI; low risk of bias was considered if > 60% of Yes on JBI

### Characteristics of included articles

Study characteristics are summarized in Tables 2 and 3. Three studies were prospective cohort studies [13–15], and five studies were retrospective cohort studies [12, 13, 16, 17]. All studies included adult patients. Concerning the type of surgery/surgical specialty, four studies addressed hysterectomy (gynaecologic surgery) [13, 14, 18], one addressed cholecystectomy [12], one addressed prostatectomy [19], and the remaining three referred to multiple abdominal surgeries [12, 16, 17]. All studies reported different surgical approaches'environmental impact using CO_2e_ [12–14, 16, 18] or CO_2_ [17]. Due to significant methodological heterogeneity, quantitative analysis was not performed.

**Table 2:** Selected studies—study design, methodology, main aim, timeline, number of surgeries evaluated and inclusion criteria

Study ID		Study Design				
Author	Year	Method	Aim	Study duration	Number of patients	Surgical approach
Gilliam et al [16]	2008	Unicentric, retrospective, cohort study	‘To estimate the effect that the expansion of laparoscopic surgery has had on global Warming’	10 years, consecutive	Unclear	Laparoscopic procedures
Power et al [17]	2012	Multicentric, retrospective, cohort study	‘To quantify the carbon footprint of Minimally Invasive Surgery (MIS)’	1 year, consecutive	2.520.223 surgeries	Minimally invasive (laparoscopic)
Cassandra et al	2014	Unicentric, prospective, cohort study	‘To evaluate emissions resulting from surgery’	1 year, consecutive	62 surgeries	Vaginal, laparotomy, laparoscopy, robotics
Woods et al [18]	2015	Unicentric, retrospective, cohort study	‘To quantify the carbon footprint of the procedures based on their energy consumed and waste produced’	4 years, consecutive	150 surgeries	Open, laparoscopic, robotics
Cassandra et al [14]	2018	Unicentric prospective, cohort study	‘To determine the carbon footprint of various sustainability interventions used for laparoscopic hysterectomy’	Monthly, consecutive	17 surgeries	Laparoscopic
Rizan et al [12]	2021	Unicentric, observational study	‘To compare the environmental and financial life cycle cost of currently available hybrid instruments for laparoscopic cholecystectomy and compared these to single-use equivalents’	N/A	N/A	Laparoscopic
Pastore et al [19]	2022	Multicentric, retrospective, cohort study	“ assessed and compared the CO2 emissions between Robot-assisted (RALP) and Laparoscopic Radical Prostatectomy (LRP)”	5 years, consecutive	223 patients	Laparoscopic, Robotic
Ramani et al	2023	Unicentric prospective, cohort study	“ determine carbon dioxide (CO2) emissions generated from nonreusable waste and compare across all types of hysterectomies”	Monthly, Consecutive (9 Months)	100 patients	Vaginal, Laparotomy, Laparoscopic, Robotic

^*^Non-applicable

**Table 3:** Selected studies, results and main conclusions

Study ID		Results			Main Conclusions
Author	Year	Type of surgery/Speciality	Environmental impact metric	Outcome	Conclusion
Gilliam et al [16]	2008	All specialties	CO_2_ used on MIS*: Carbon Footprint CO_2e_	Each CO_2_ cylinder produces *0.9 kgCO*_*2e*_	‘Despite increasing frequency of the laparoscopic approach in general surgery, its impact on global warming is negligible.’
*Power *et al [17]	2012	All specialties	CO_2_ used for MIS*	CO_2_ emissions from MIS*: *355.924 tones/year* (estimated)	‘The CO_2_ emissions of MIS in the United States have a significant environmental impact. This should be considered in larger strategies to reduce healthcare’s carbon footprint while maximizing healthcare quality.’
Cassandra et al	2014	Gynaecology-Hysterectomy	Life Cycle Assessment (Waste & Process)	Minimally invasive surgery presents higher footprint	‘Across the whole healthcare industry, there is a profound opportunity to make healthcare services more efficient environmentally and economically without compromising safety or efficacy’
Woods et al [18]	2015	Gynaecology-Hysterectomy	Waste and energy converted to CO_2e_	Robotics: *40.3 kgCO*_*2e*_ Laparoscopic: *29.2 kgCO*_*2e*_ Open: *22.7 kgCO*_*2e*_	‘Our results provide clinicians, administrators and policymakers with knowledge of the environmental impact of their decisions to facilitate adoption of sustainable practices.’
Cassandra et al [14]	2018	Gynaecology-Hysterectomy	Hybrid environmental life cycle assessment CO_2e_	Laparoscopic hysterectomy *562 kgCO*_*2e*_ (per procedure)	‘To reduce the environmental emissions of surgeries, health care providers need to implement a combination of approaches, including minimizing materials, moving away from certain heat-trapping anesthetic gases, maximizing instrument reuse or single-use device reprocessing and reducing off-hour energy use in the operating room These strategies can reduce the carbon footprint of an average laparoscopic hysterectomy by up to 80%.’
Rizan et al [12]	2021	General SurgeryCholecystectomy	Life Cycle Assessment of the single-use and non-single-use (Hybrid) CO_2e_	Hybrid: *1.756 kgCO*_*2e*_ Single use: *7.194 kgCO*_*2e*_	‘The carbon footprint of using hybrid instruments for laparoscopic cholecystectomy is around a quarter of that for single use equivalents and the financial cost around half… adoption of hybrid instruments could play an important role in meeting carbon reduction targets in healthcare…’
Pastore et al [19]	2022	Urology–Prostatectomy	Life Cycle Assessment of surgical devices, Energy consumption & Hospital Stay CO_2e_	Laparoscopic: *59.7 kgCO*_*2e*_ Robotic: *47.3 kgCO*_*2e*_	“robot-assisted radical prostatectomy generates substantially less CO2 per procedure than laparoscopic prostatectomy because of the use of more reusable surgical supplies and shorter operative time and hospital stays, resulting in a lower environmental impact.”
Ramani et al	2023	Gynaecology—Hysterectomy	Waste converted to CO_2e_	Vaginal: *4.2 kgCO*_*2e*_ Laparotomy: *7.1 kgCO*_*2e*_ Laparoscopic: *10.7 kgCO*_*2e*_ Open: *12.0 kgCO*_*2e*_	“ Robotic hysterectomies generated a statistically significant majority of CO2 emissions. Therefore, robotic surgery, as currently practiced, may offer a good initial opportunity for decreasing the carbon footprint of surgery”

^*^MIS Minimally invasive surgery

#### Outcomes

### Environmental impact of different surgical approaches

Only two studies compared the carbon footprint between open, laparoscopic and robotic approaches [18]. The first one, a retrospective cohort study, evaluated the environmental impact of waste generation and energy dispended during hysterectomy for endometrial cancer. These indicators were converted to CO_2e_ for the presentation of the results. After assessing 50 procedures by each approach, the authors concluded that an open hysterectomy generated 22.7 kgCO_2e_ of waste and energy. The same procedure performed by laparoscopy or robotics produced 29.2 kgCO_2e_ or 40.3 kgCO_2e,_ respectively. The second was a unicentric prospective cohort study that evaluated the waste environmental impact (converted to CO_2e_) generated for all the abdominal approaches on Hysterectomy surgery (independently of the indication). Robotic hysterectomies generated a statistically significant amount of CO2 emissions (12.0 kgCO_2e_) [15] (Table 3).

Two other studies on hysterectomy focused on the waste generated by different surgical approaches (vaginal, open, laparoscopy, and robotics). Both studies concluded that minimally invasive surgery presented a higher waste carbon footprint[13]. However, concerning for laparoscopic hysterectomy, the carbon footprint could be reduced by up to 80% by adopting a combination of measures [14] (Table 3)**.**

One recent retrospective multicentric cohort study evaluated the carbon footprint of minimally invasive prostatectomy surgery (laparoscopic vs robotic). The authors assessed the life cycle assessment of surgical devices and energy consumption. Additionally, the length of the hospital stay was also considered. This throughout analysis concluded that"robot-assisted radical prostatectomy generated substantially less CO2 per procedure than laparoscopic prostatectomy (47.3 vs 59.7 kgCO_2e_, respectively)[19].

### Environmental impact of single-use and non-single-use instruments

Rizan et al*.* assessed the environmental burden and financial costs of currently available hybrid instruments (non-single-use) for laparoscopic cholecystectomy [12]. These devices were compared to single-use equivalents. The non-single-use devices generated a lower carbon footprint per procedure (1,756 kgCO_2e_ *vs* 7,194 kgCO_2e;_) [12] (Table 3).

### Environmental impact of carbon dioxide used for pneumoperitoneum

Power et al*.* and Gilliam et al*.* discussed the environmental impact of CO_2_ used in minimally invasive surgery [16, 17]. Power et al*.* assessed the number of minimally invasive surgeries performed for one year in the United States of America in 2010, and estimated that approximately 355,924 tonnes of CO_2_ emissions were due to CO_2_ used in minimally invasive surgery[17]. Similarly, Gilliam et al. determined the use of CO_2_ in minimally invasive surgery, in a single hospital, for 10 years. However, this study assessed other indicators, such as the impact of the surgical procedure duration. The median operative time for the laparoscopic procedures was 1.01 h (range 0.3–4.45 h), with an operative time per cylinder of 3.96 h. Each carbon dioxide cylinder produced a carbon footprint of 0.9 kgCO_2e_ but could be used in more than one surgery. The authors concluded that the impact of CO_2_ use per procedure was negligible in minimally invasive surgery [16].

Qualitative subgroup analysis.

Due to methodological diversity, we conducted a qualitative subgroup analysis based on the type of abdominal surgery. For hysterectomy, four studies consistently showed minimally invasive techniques (laparoscopic and robotic) produced higher CO2e compared to open procedures, mainly due to increased disposable instrument use [13–15, 18]. In prostatectomy, however, robotic surgery demonstrated lower overall emissions compared to laparoscopy due to fewer disposable instruments, shorter hospital stays, and reduced operating room time [19]. Another qualitative subgroup analysis based on the carbon footprint measurement methodologies revealed that comprehensive life-cycle assessments resulted in considerably higher CO2e estimates (e.g., 562 kgCO2e) compared to those assessing only intraoperative waste and energy use (e.g., 29.2 kgCO2e), highlighting the critical importance of assessment methods on reported environmental impacts [14, 18].

### Clinical outcomes

All studies were systematically reviewed for clinical outcomes. However, only one study considered the length of hospital stay as a clinical outcome [19]. Therefore, no specific analysis was performed regarding patients’ clinical outcomes.

## Discussion

This systematic review found substantial methodological heterogeneity among the included studies, notably related to the types of surgical procedures analyzed, methods of carbon footprint calculation, and environmental assessment techniques employed. For instance, the studies varied significantly in their scope, some considered only waste and energy consumption during surgery, while others included broader life-cycle assessments covering multiple phases of patient care [14, 18, 19]. Additionally, the considerable variation in CO_2e_ values reported, such as the discrepancy between the carbon footprint of laparoscopic hysterectomy reported by *Cassandra *et al. (562 kgCO_2e_) and *Woods *et al. (29.2 kgCO_2e_), further underscores these methodological differences. Although this variability inherently limits the generalizability and strength of our synthesized conclusions, it also underscores the critical need for systematic reviews such as this one to clearly identify existing methodological gaps. Indeed, without systematically reviewing available evidence, it would remain unclear how the heterogeneity in previous studies might influence our understanding of such an important and timely topic as sustainability in surgery. Therefore, despite the identified heterogeneity and necessary caution in interpreting results, the value of this systematic review remains high, serving as a foundation and roadmap for more robust, standardized future research. Importantly, this systematic review did not involve direct research with human participants or animals. Clinical outcomes weren't evaluated, and thus, ethical approval and informed consent were not applicable. However, future research building upon this review should prioritise ethical rigour, especially when prospectively collecting data on sustainability-related interventions from patients or surgical teams and clinical outcomes. 

Our review suggests that minimally invasive surgery has a higher carbon footprint when compared to open surgery. Variables such as indicators used to estimate surgery’s carbon footprint, and differences in timelines limit the generalizability of results. Notably, only *Woods* et al*.* and *Ramani *et al*.* assessed the carbon footprint of open surgery, reducing available comparative [15, 18]. Moreover, patient outcome advantages of minimally invasive techniques were largely unaccounted for, except in the study by Pastore et al., where robotic prostatectomy had a lower environmental impact compared to laparoscopic surgery, primarily due to fewer disposables, shorter operative time, and reduced hospital stays [19]. On the other hand, a recent systematic review on environmental sustainability in robotic and laparoscopic surgery concluded that robotic procedures resulted in the highest greenhouse gases emissions and waste production.

This systematic review focused not only on abdominal surgery, but also on ophthalmologic surgery. This work underlined that multiple alternatives could be used to reduce the carbon footprint of minimally invasive surgery, such as using “reusable equipment, repackaging, surgeon preference cards, increasing staff awareness on open and unused equipment and desflurane avoidance” [20]. This underscores the importance of evaluating clinical outcomes throughout the patient pathway to fully appreciate the environmental impact of surgical procedures. Potential benefits of minimally invasive surgery such as reduced inpatient days, lower medication usage for pain control, and decreased complications (e.g., wound infection, incisional hernias) could further diminish its environmental footprint when fully assessed [21].

Gilliam et al*.* and Power et al*.* focused on the carbon dioxide used for abdominal surgery pneumoperitoneum [16, 17]. During abdominal minimally invasive surgery (laparoscopic or robotic), the abdominal cavity is inflated with gas (CO_2_) to create space to examine the surgical site and manipulate instruments. Thus, Woods et al*.* and Power et al. elaborated on the CO_2_ carbon footprint during minimally invasive surgery [16, 17]. They concluded that the overall consumption of CO_2_ per minimally invasive surgery seems negligible [16]. Indeed, considering CO_2_ as the only difference between open and minimally invasive abdominal surgery would be reductive.

Minimally invasive surgery also comes along with a broad collection of instruments to be used [5]. Technological advances have revolutionized surgical procedures, and increasingly sophisticated and refined devices have been developed for laparoscopic and robotic procedures. There is an investment trend towards disposable rather than reusable instruments [22]. As expected, Rizan et al*.* concluded that single-use instruments, compared to non-single-use, resulted in a 6-time higher carbon footprint for laparoscopic cholecystectomy [12]. Single-use devices generate not only more waste per surgery, but these results are also reproduced in the long-term[22, 23]. Both disposable and reusable instruments are considered safe, underscoring the potential for environmental-based decision-making to meaningfully reduce surgery’s carbon footprint without compromising patient safety [22].

This systematic review's comprehensive search strategy, incorporating diverse databases and both retrospective and prospective cohort studies, thoroughly explores the environmental impact of abdominal surgeries. The review offers valuable insights into the carbon footprint of different techniques by including studies on various surgical approaches and settings. It also emphasizes the need to consider patient pathways and clinical outcomes alongside carbon emissions, enhancing the understanding of surgical sustainability.

However, the review faced methodological heterogeneity, particularly in carbon footprint assessments and reporting standards, which limited the ability to perform quantitative comparisons. Qualitative subgroup analyses further underscored substantial methodological diversity and heterogeneity, particularly when stratified by type of abdominal surgery (e.g., hysterectomy versus prostatectomy) and carbon footprint measurement methodologies. Again, these subgroup findings emphasize the necessity of careful interpretation of pooled results and underline the significant influence of methodological choices on reported environmental impacts.

To enhance the accuracy and comparability of future studies, it would be beneficial for future research to consider adopting standardized life-cycle assessment frameworks that incorporate comprehensive environmental impact measures across the entire patient journey. Implementing consistent metrics for reporting carbon footprint outcomes, such as universally agreed-upon systems for quantifying surgical waste, instrument disposability, energy consumption, and patient outcomes (hospital stays, medication usage, complications), could help reduce methodological biases. Such standardized approaches may facilitate more definitive and reliable conclusions about the environmental sustainability of surgical techniques in future systematic reviews.

## Conclusion

This systematic review suggests that minimally invasive surgeries, particularly robotic and laparoscopic techniques, generally have a higher carbon footprint compared to open surgery, primarily due to the use of disposable instruments and waste generation. However, the environmental impact of these procedures cannot be fully understood without considering the broader clinical outcomes and patient pathways. Notably, robotic prostatectomy demonstrated a lower overall carbon footprint when factors such as shorter hospital stays and reduced operative times were accounted for. Due to the substantial methodological heterogeneity identified, definitive conclusions remain limited. Future research should prioritize standardized environmental assessment methods, comprehensive life-cycle analyses and clinical outcomes to reliably clarify the environmental sustainability of different surgical techniques.

## Supplementary Information

Below is the link to the electronic supplementary material.

Supplementary file1 (DOCX 16 KB)

## Data Availability

All data generated or analysed during this study are included in the supplementary material, which provides the full search strings and detailed methodology used in the review.
