# Supplementary material for: Green surgery: a systematic review of the environmental impact of laparotomy, laparoscopy, and robotics
Source: Updates Surg. 2025 May 21;77(5):1683–92. doi: 10.1007/s13304-025-02221-1 (PMC12420719; doi:10.1007/s13304-025-02221-1)
Supplement: Supplementary file 1 — Supplementary file1 (DOCX 16 KB) [file 13304_2025_2221_MOESM1_ESM.docx]

**Laparotomy, Laparoscopy or robotics: What is the most sustainable surgical approach - Systematic Review**

**Question:**

In Surgery, what is the most sustainable surgical approach, by laparotomy by laparoscopy or robotics?

P: Surgeries / Surgical interventions (abdominal)

I: Laparotomy Surgery

C: Laparoscopic, robotics Surgery

O: Environmental sustainability

Measure: mass of carbon dioxide (kg CO(2) e) release into the environment

**Databases:**

Pubmed

Embase / Scopus

**String and Mesh Terms:**

***Surgery***

**Surgery** [5,057,023](https://pubmed.ncbi.nlm.nih.gov/?term=Surgery&sort=&size=200)

"surgery"[MeSH Subheading] OR "surgery"[All Fields] OR "surgical procedures, operative"[MeSH Terms] OR ("surgical"[All Fields] AND "procedures"[All Fields] AND "operative"[All Fields]) OR "operative surgical procedures"[All Fields] OR "general surgery"[MeSH Terms] OR ("general"[All Fields] AND "surgery"[All Fields]) OR "general surgery"[All Fields] OR "surgery s"[All Fields] OR "surgerys"[All Fields] OR "surgeries"[All Fields]

***Approach:***

**(((Laparotomy Surgery) OR (Laparoscopic Surgery))) OR (Robotic surgery)** [198,879](https://pubmed.ncbi.nlm.nih.gov/?term=%28%28%28Laparotomy+Surgery%29+OR+%28Laparoscopic+Surgery%29%29%29+OR+%28Robotic+surgery%29&sort=&size=200)

(("laparotomy"[MeSH Terms] OR "laparotomy"[All Fields] OR "laparotomies"[All Fields]) AND ("surgery"[MeSH Subheading] OR "surgery"[All Fields] OR "surgical procedures, operative"[MeSH Terms] OR ("surgical"[All Fields] AND "procedures"[All Fields] AND "operative"[All Fields]) OR "operative surgical procedures"[All Fields] OR "general surgery"[MeSH Terms] OR ("general"[All Fields] AND "surgery"[All Fields]) OR "general surgery"[All Fields] OR "surgery s"[All Fields] OR "surgerys"[All Fields] OR "surgeries"[All Fields])) OR ("laparoscopy"[MeSH Terms] OR "laparoscopy"[All Fields] OR ("laparoscopic"[All Fields] AND "surgery"[All Fields]) OR "laparoscopic surgery"[All Fields]) OR ("robotic surgical procedures"[MeSH Terms] OR ("robotic"[All Fields] AND "surgical"[All Fields] AND "procedures"[All Fields]) OR "robotic surgical procedures"[All Fields] OR ("robotic"[All Fields] AND "surgery"[All Fields]) OR "robotic surgery"[All Fields])

***Sustainability:***

**(Sustainability) OR (Carbon Footprint)) OR (Environmental Sustainability)** 4,143

"sustain"[All Fields] OR "sustainability"[All Fields] OR "sustainable"[All Fields] OR "sustainably"[All Fields] OR "sustained"[All Fields] OR "sustaining"[All Fields] OR "sustainment"[All Fields] OR "sustains"[All Fields] OR ("carbon footprint"[MeSH Terms] OR ("carbon"[All Fields] AND "footprint"[All Fields]) OR "carbon footprint"[All Fields]) OR (("environment"[MeSH Terms] OR "environment"[All Fields] OR "environmental"[All Fields] OR "environmentally"[All Fields] OR "environmentals"[All Fields]) AND ("sustain"[All Fields] OR "sustainability"[All Fields] OR "sustainable"[All Fields] OR "sustainably"[All Fields] OR "sustained"[All Fields] OR "sustaining"[All Fields] OR "sustainment"[All Fields] OR "sustains"[All Fields]))

***String:***

**((Surgery) AND ((((Laparotomy Surgery) OR (Laparoscopic Surgery))) OR (Robotic surgery))) AND (((Sustainability) OR (Carbon Footprint)) OR (Environmental Sustainability)) *1740***

("surgery"[MeSH Subheading] OR "surgery"[All Fields] OR "surgical procedures, operative"[MeSH Terms] OR ("surgical"[All Fields] AND "procedures"[All Fields] AND "operative"[All Fields]) OR "operative surgical procedures"[All Fields] OR "general surgery"[MeSH Terms] OR ("general"[All Fields] AND "surgery"[All Fields]) OR "general surgery"[All Fields] OR "surgery s"[All Fields] OR "surgerys"[All Fields] OR "surgeries"[All Fields]) AND ((("laparotomy"[MeSH Terms] OR "laparotomy"[All Fields] OR "laparotomies"[All Fields]) AND ("surgery"[MeSH Subheading] OR "surgery"[All Fields] OR "surgical procedures, operative"[MeSH Terms] OR ("surgical"[All Fields] AND "procedures"[All Fields] AND "operative"[All Fields]) OR "operative surgical procedures"[All Fields] OR "general surgery"[MeSH Terms] OR ("general"[All Fields] AND "surgery"[All Fields]) OR "general surgery"[All Fields] OR "surgery s"[All Fields] OR "surgerys"[All Fields] OR "surgeries"[All Fields])) OR ("laparoscopy"[MeSH Terms] OR "laparoscopy"[All Fields] OR ("laparoscopic"[All Fields] AND "surgery"[All Fields]) OR "laparoscopic surgery"[All Fields]) OR ("robotic surgical procedures"[MeSH Terms] OR ("robotic"[All Fields] AND "surgical"[All Fields] AND "procedures"[All Fields]) OR "robotic surgical procedures"[All Fields] OR ("robotic"[All Fields] AND "surgery"[All Fields]) OR "robotic surgery"[All Fields])) AND ("sustain"[All Fields] OR "sustainability"[All Fields] OR "sustainable"[All Fields] OR "sustainably"[All Fields] OR "sustained"[All Fields] OR "sustaining"[All Fields] OR "sustainment"[All Fields] OR "sustains"[All Fields] OR ("carbon footprint"[MeSH Terms] OR ("carbon"[All Fields] AND "footprint"[All Fields]) OR "carbon footprint"[All Fields]) OR (("environment"[MeSH Terms] OR "environment"[All Fields] OR "environmental"[All Fields] OR "environmentally"[All Fields] OR "environmentals"[All Fields]) AND ("sustain"[All Fields] OR "sustainability"[All Fields] OR "sustainable"[All Fields] OR "sustainably"[All Fields] OR "sustained"[All Fields] OR "sustaining"[All Fields] OR "sustainment"[All Fields] OR "sustains"[All Fields])))

**Translations**

**Surgery:** "surgery"[Subheading] OR "surgery"[All Fields] OR "surgical procedures, operative"[MeSH Terms] OR ("surgical"[All Fields] AND "procedures"[All Fields] AND "operative"[All Fields]) OR "operative surgical procedures"[All Fields] OR "general surgery"[MeSH Terms] OR ("general"[All Fields] AND "surgery"[All Fields]) OR "general surgery"[All Fields] OR "surgery's"[All Fields] OR "surgerys"[All Fields] OR "surgeries"[All Fields]

**Laparotomy:** "laparotomy"[MeSH Terms] OR "laparotomy"[All Fields] OR "laparotomies"[All Fields]

**Surgery:** "surgery"[Subheading] OR "surgery"[All Fields] OR "surgical procedures, operative"[MeSH Terms] OR ("surgical"[All Fields] AND "procedures"[All Fields] AND "operative"[All Fields]) OR "operative surgical procedures"[All Fields] OR "general surgery"[MeSH Terms] OR ("general"[All Fields] AND "surgery"[All Fields]) OR "general surgery"[All Fields] OR "surgery's"[All Fields] OR "surgerys"[All Fields] OR "surgeries"[All Fields]

**Laparoscopic Surgery:** "laparoscopy"[MeSH Terms] OR "laparoscopy"[All Fields] OR ("laparoscopic"[All Fields] AND "surgery"[All Fields]) OR "laparoscopic surgery"[All Fields]

**Robotic surgery:** "robotic surgical procedures"[MeSH Terms] OR ("robotic"[All Fields] AND "surgical"[All Fields] AND "procedures"[All Fields]) OR "robotic surgical procedures"[All Fields] OR ("robotic"[All Fields] AND "surgery"[All Fields]) OR "robotic surgery"[All Fields]

**Sustainability:** "sustain"[All Fields] OR "sustainability"[All Fields] OR "sustainable"[All Fields] OR "sustainably"[All Fields] OR "sustained"[All Fields] OR "sustaining"[All Fields] OR "sustainment"[All Fields] OR "sustains"[All Fields]

**Carbon Footprint:** "carbon footprint"[MeSH Terms] OR ("carbon"[All Fields] AND "footprint"[All Fields]) OR "carbon footprint"[All Fields]

**Environmental:** "environment"[MeSH Terms] OR "environment"[All Fields] OR "environmental"[All Fields] OR "environmentally"[All Fields] OR "environmentals"[All Fields]

**Sustainability:** "sustain"[All Fields] OR "sustainability"[All Fields] OR "sustainable"[All Fields] OR "sustainably"[All Fields] OR "sustained"[All Fields] OR "sustaining"[All Fields] OR "sustainment"[All Fields] OR "sustains"[All Fields]
